# Dimethoxycurcumin Acidifies Endolysosomes and Inhibits SARS-CoV-2 Entry

**DOI:** 10.3389/fviro.2022.923018

**Published:** 2022-06-29

**Authors:** Nabab Khan, Zahra Afghah, Aparajita Baral, Jonathan D. Geiger, Xuesong Chen

**Affiliations:** Department of Biomedical Sciences, University of North Dakota School of Medicine and Health Sciences, Grand Forks, ND, United States

**Keywords:** dimethoxycurcumin, ACE2 (angiotensin converting enzyme-2), lysosome, acidification, SARS-CoV-2

## Abstract

The pandemic of coronavirus disease 2019 (COVID-19) caused by infection by severe acute respiratory syndrome-coronavirus-2 (SARS-CoV-2) continues to take a huge toll on global health. Although improving, currently there are only limited therapies against SARS-CoV-2. Curcumin, a natural polyphenol, exerts antiviral effects against a wide variety of viruses and can inhibit SARS-CoV-2 entry. However, undesirable physicochemical and pharmacokinetic properties of curcumin limit its clinical application. Here, we determined the effects of dimethoxycurcumin (DiMC), a methylated analog of curcumin with improved bioavailability, on the entry of SARS-CoV-2. DiMC blocked entry of pseudo-SARS-CoV-2 into Calu-3 human non-small cell lung adenocarcinoma cells and Vero E6 green monkey kidney epithelial cells. Mechanistically, DiMC acidified lysosomes, enhanced lysosome degradation capabilities, and promoted lysosome degradation of angiotensin converting enzyme 2 (ACE2), a major receptor for SARS-CoV-2 entry, as well as pseudo-SARS-CoV-2 and the SARS-CoV-2 S1 protein. Furthermore, other lysosome acidifying agents, including the TRPML1 agonist ML-SA1 and the BK channel activator NS1619, also blocked the entry of pseudo-SARS-CoV-2. Thus, the anti-SARS-CoV-2 potential of DiMC and lysosome acidifying agents might be explored further as possible effective therapeutic strategies against COVID-19.

## INTRODUCTION

Infection by severe acute respiratory syndrome-coronavirus-2 (SARS-CoV-2) causes the current pandemic of coronavirus disease 2019 (COVID-19) (1, 2). COVID-19 has resulted in over 200 million confirmed cases and almost 4.7 million deaths (https://www.who.int/emergencies/diseases/novel-coronavirus-2019). SARS-CoV-2 is an enveloped single-stranded RNA virus, and like other enveloped viruses, SARS-CoV-2 enters host cells and utilizes host cell machinery for replication. SARS-CoV-2 enters the host cell by either direct fusion of the viral envelope with plasma membranes of host cells or fusion with endosomes following endocytosis (3–6). Once fused with host cell membranes, viral RNA is released into the cytosol where viral replication occurs (7, 8).

Although improving, there are limited drugs and therapeutic strategies to prevent SARS-CoV-2 infection and to combat COVID-19. Curcumin, a natural polyphenol derived from the Indian spice turmeric, has been reported to exert antiviral effects against a wide variety of viruses including influenza A, dengue, zika, hepatitis C, HIV-1, and SARS-CoV (9). Recent findings from *in silico* modelling (10, 11) and *in vitro* studies (12) indicate that curcumin can disrupt SARS-CoV-2 spike protein-mediated receptor-binding and inhibit cellular entry of SARS-CoV-2. However, curcumin is metabolically unstable, has low water solubility, and has poor bioavailability; physicochemical and pharmacokinetic properties that limit its clinical application (13, 14).

As a methylated analog of curcumin (13, 15), dimethoxycurcumin (DiMC) is more metabolically stable, has better bioavailability, and exerts greater antioxidant (16) and anti-inflammatory properties (17) than does curcumin. Here, we determined effects of DiMC on the entry of SARS-CoV-2 pseudo-virus and found that DiMC blocked the entry of SARS-CoV-2 pseudo-virus in Calu-3 human non-small cell lung adenocarcinoma cells and Vero E6 green monkey kidney epithelial cells, acidified lysosomes, enhanced lysosome degradation capabilities, and promoted lysosome degradation of ACE2 as well as pseudo-SARS-CoV-2 and the SARS-CoV-2 S1 protein. Furthermore, other lysosome acidifying agents also blocked the SARS-CoV-2 S protein-mediated entry of pseudo-SARS-CoV-2. Thus, the anti-SARS-CoV-2 potential of DiMC and lysosome acidifying agents might be explored further as possible effective therapeutic strategies against COVID-19.

## MATERIAL AND METHODS

### Cell Culture

Human non-small cell lung adenocarcinoma Calu-3 cells and green monkey kidney epithelial Vero E6 cells were purchased from ATCC and cultured in 1X EMEM (Calu-3) and DMEM (Vero E6) supplemented with 10% fetal bovine serum (FBS) and 1X penicillin and streptomycin antibiotic at 37°C in 5% CO_2_ incubator. For our experiments, cells were not used after 10 passages.

### Cellular Entry of SARS-CoV-2 Spike Protein Pseudo-Virus

Following pretreatment with DiMC, ML-SA1, NS1619, or DMSO as a control for 18 hr, Calu-3 and VeroE6 cells cultured on 96-well plates were treated with luciferase-integrated and SARS-CoV-2 spike protein-conjugated pseudovirus for 6 h, washed 3-times with media, and incubated for another 36 h according to the manufacturer protocol (BPS Biosciences). Post-incubation, S protein-mediated entry of SARS-CoV-2 pseudo virus was estimated by luciferase activity, which was determined with a steady glow luciferase assay (Promega). Luciferase activity was measured as luminescence relative light unit (RLU) using microplate reader (Synergy H1).

### Cathepsin B Activity Assay

Magic Red was used to measure cathepsin B activity. Magic Red substrate is cleaved by active cathepsins and Magic Red cresyl violet fluorescence was measured by confocal microscopy (Zeiss LSM 800). Cells at 30 to 40% confluency (~10K cells) were seeded were seeded on 35 mm^2^ dishes and incubated with Magic Red at a 1:500 dilution and Hoechst 33342 (nucleus stain) for 30 minutes. Cells were washed three-times with PBS and fluorescence images were acquired by confocal microscopy; excitation/emission wavelengths were 592/628 and data were analyzed using ImageJ software.

### Lysosome pH Assay

Endolysosome pH was measured using LysoBrite Green (AAT Bioquest); a pH-sensitive dye that selectively accumulates in lysosomes *via* the lysosomal pH gradient. Cells at 30 to 40% confluency (~10K cells) were seeded on 35 mm^2^ dishes and incubated for 40 min at 37°C with LysoBrite green at a 1:500 dilution. Cells were washed three times with PBS and fluorescence images were acquired at an excitation of 543 nm and emission of 565 nm by confocal microscopy (Zeiss LSM 800). Data were analyzed using ImageJ software.

### Immunoblotting

Cells receiving various treatments were harvested and lysed in 1 × RIPA lysis buffer (Thermo Fisher) containing 10 mM NaF, 1 mM Na_3_VO_4_, and 1 × protease inhibitor cocktail (Sigma). After centrifugation (14,000 × g for 15 min at 4°C), supernatants were collected, and protein concentrations were determined with a Bradford protein assay (Bio-Rad). SDS-PAGE (4–12% gel) was used to separate proteins (10 μg/lane) and blots were transferred to nitrocellulose membranes using the iBlot 2 dry transfer system (Invitrogen). Membranes were incubated overnight at 4°C with antibodies against ACE2 (SinoBiological), SARS-CoV-2 S1 (GenScript), SARS-CoV-2 S2 (GeneTex, 1A9), ATG5 (Abcam), LC3B (Sigma), actin (Abcam), and GAPDH (Abcam). Blots were developed with enhanced chemiluminescence, and the density of antibody-positive protein bands was determined using an Odyssey Fc Imaging System (LiCor).

### Immunostaining

Calu-3 cells were fixed with 4% paraformaldehyde for 5 min followed by cold methanol (−20°C) for 15 min. Following washing and blocking with 5% BSA, cells were incubated overnight at 4°C with primary antibodies (1:100) against SARS-CoV-2 S2 protein (GeneTex, 1A9). Cells were then washed with PBS and incubated with corresponding Alexa 647-conjugated secondary antibodies (Invitrogen). Cells were examined by Zeiss LSM800 confocal microscopy and data were analyzed by ImageJ software.

### Spike Protein Internalization Assay

Calu-3 cells pretreated with DiMC (1.0, 2.0, and 4.0 μM for 24 h) were incubated with SARS-CoV-2 S1 protein (5.0 μg/ml, GenScript) for an additional period of 6 h. Following washing, cells were harvested for immunoblotting to determine cellular levels of internalized SARS-CoV-2 S1 protein.

### Cell Toxicity Assay

Cell toxicity was quantitatively assessed by the measurement of lactate dehydrogenase (LDH) released from damaged or destroyed cells into the extracellular fluid (Pierce). Cells were treated with DiMC (dissolved in DMSO) for 24 hr. DMSO was used as a vehicle control and a positive control supplied by Pierce was used. Following treatment, an aliquot (50 μL) of bathing media was combined with NADH and pyruvate solutions. LDH activity is proportional to the rate of pyruvate loss, which was assayed by absorbance change using a microplate reader (Synergy H1). Data were expressed as percentages of positive control.

### Statistical Analysis

All data were presented as means ± standard deviations. Statistical significance between two groups was determined with Student’s t-test, and statistical significance among multiple groups was determined using a one-way ANOVA plus a Tukey *post-hoc* test. p<0.05 was accepted to be statistically significant.

## RESULTS

### DiMC Blocked SARS-CoV-2 Spike Protein-Mediated Entry of Pseudo-SARS-CoV-2

**FIGURE 1 |:**
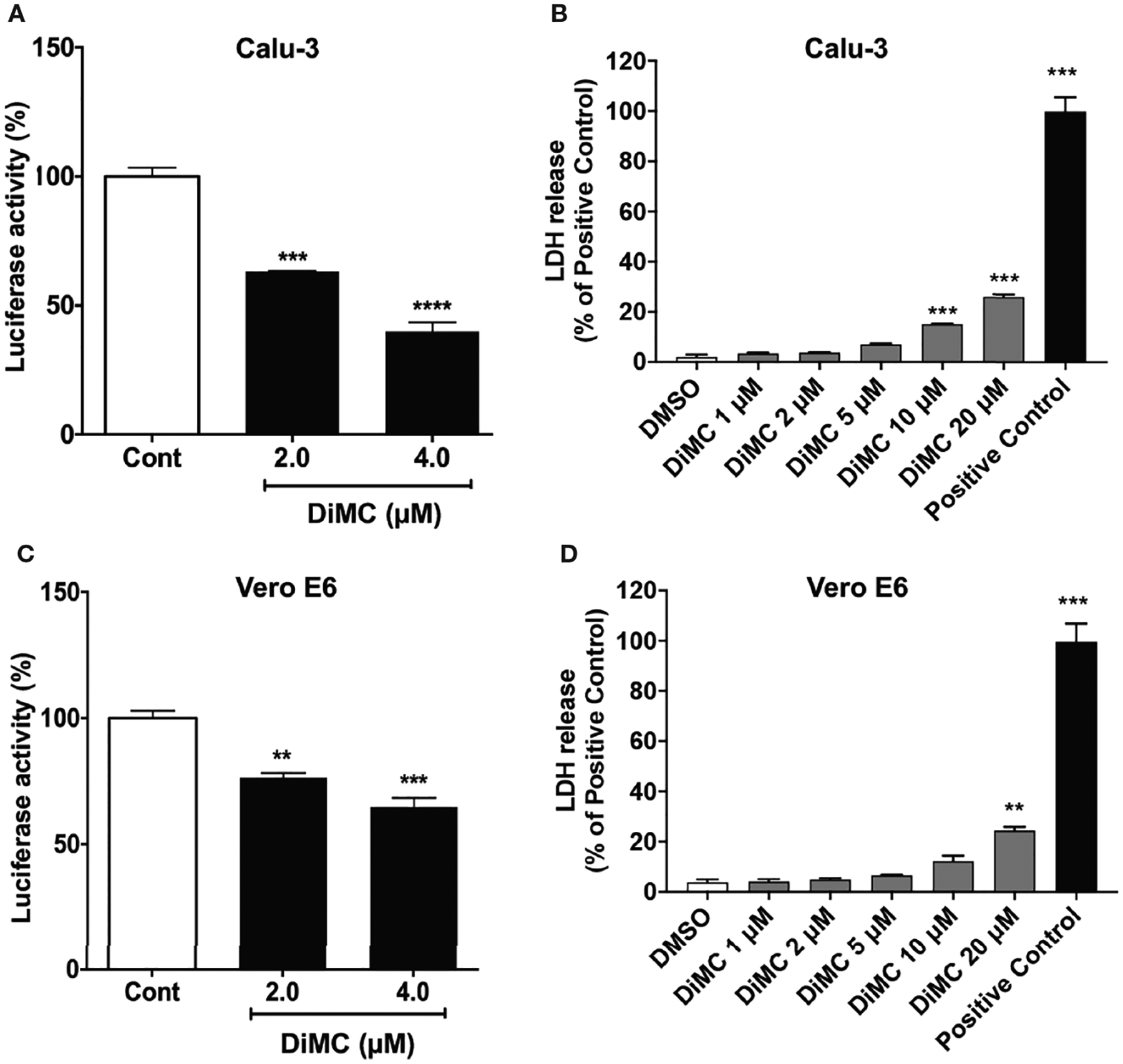
DiMC blocked SARS-CoV-2 spike protein-mediated cellular entry of pseudo-SARS-CoV-2: DiMC treatment (2.0 and 4.0 μM for 18 h) significantly attenuated SARS-CoV-2 spike protein-mediated pseudo-SARS-CoV-2 cellular entry as indicated by decreases in luciferase activity in both Calu-3 **(A)** and Vero E6 **(C)** cells (n = 3, **p <0.01, ***p <0.001, ****p <0.0001). As indicated by LDH cytotoxicity assay, DiMC at the concentration of 5 μM or lower was not toxic to Calu-3 cells **(B)** or Vero E6 cells **(D)** (n = 3, ***p <0.001).

First, we determined effects of DiMC on the SARS-CoV-2 spike protein-mediated entry of pseudo-SARS-CoV-2 using a viral entry luciferase reporter gene in Vero E6 cells derived from the kidney of an African green monkey and Calu-3 human lung cells. In both cell lines, DiMC (2 and 4 μM for 6 h) significantly attenuated spike protein-mediated pseudo-SARS-CoV-2 entry as indicated by decreases in luciferase activity (Figure 1A, C). As indicated by results from an LDH cytotoxicity assay, DiMC at the concentration of 5 μM or lower was not toxic to Calu-3 cells (Figure 1B) or to Vero E6 cells (Figure 1D). Significantly, such no-toxic concentrations (2 and 4 μM) of DiMC are achievable in mice treated with DiMC (18, 19). Because entry of SARS-CoV-2 entry into Vero E6 cells is cathepsin L-dependent and entry into Calu-3 cells is dependent on TMPRSS2 (20, 21), these findings suggest that DiMC affects SARS-CoV-2 entry *via* multiple mechanisms.

### DiMC Decreased Protein Levels of ACE2

**FIGURE 2 |:**
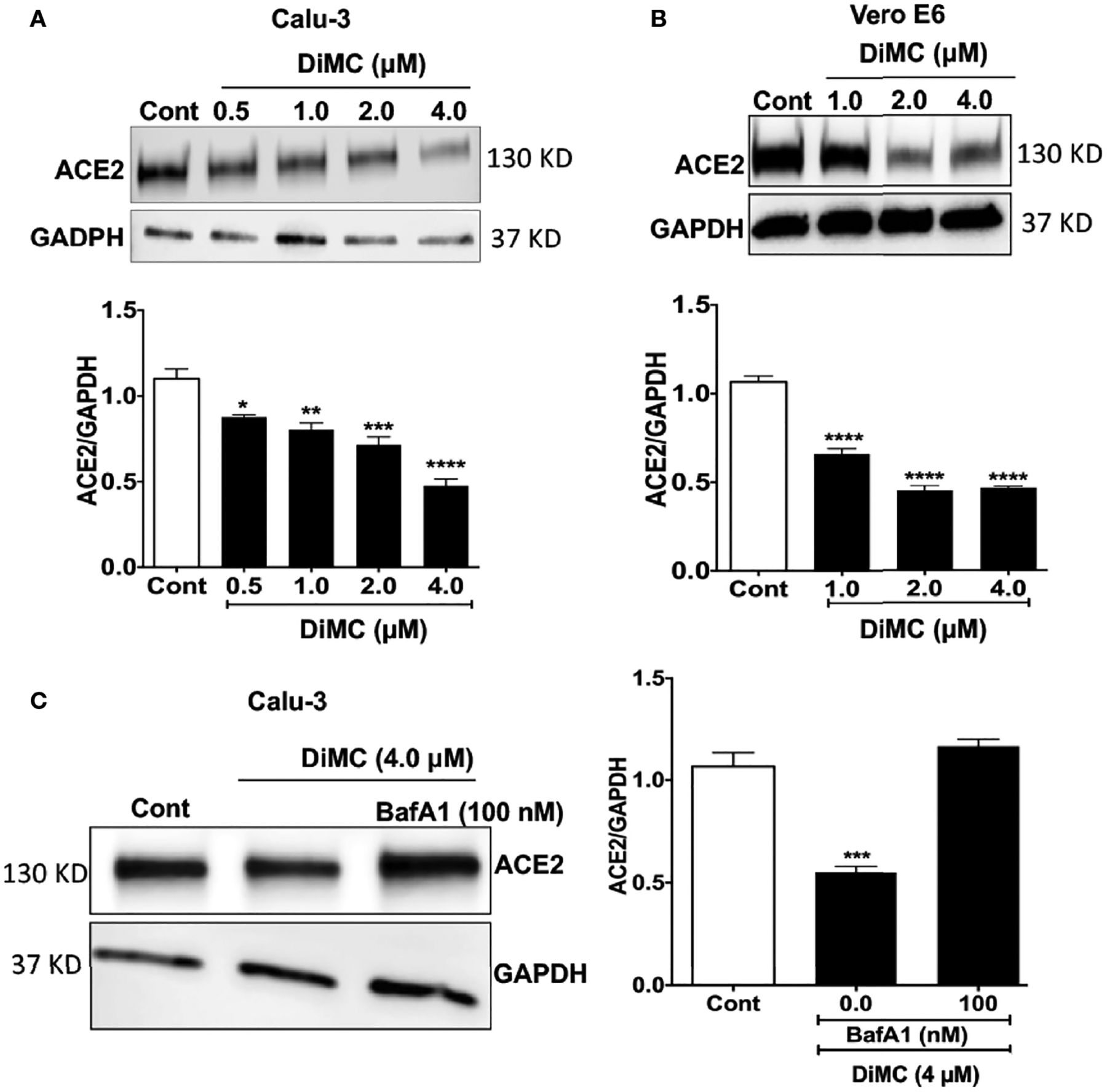
DiMC decreased protein expression levels of ACE2: **(A, B)** DiMC treatment (1.0, 2.0, and 4.0 μM for 24 h) decreased protein expression levels of ACE2 in a concentration-dependent manner in both Calu-3 **(A)** and Vero E6 **(B)** cells (n = 3, *p <0.05, **p <0.01, ***p <0.001, ****p <0.0001). **(C)** Inhibiting vacuolar-ATPase with bafilomycin A1 (100 nM) prevented DiMC (4.0 μM for 18 h)-induced decreases of ACE2 protein expression levels in Calu-3 cells (n = 3, ***p <0.001).

Because ACE2 is involved in the entry of SARS-CoV-2 into both Calu-3 and Vero E6 cells (20), next we determined effects of DiMC on protein expression levels of ACE2. In both Calu-3 cells (Figure 2A) and Vero E6 cells (Figure 2B), DiMC (0.5 – 4 μM for 24 h) significantly decreased protein expression levels of ACE2 in a concentration-dependent manner. Because the binding of SARS-CoV-2 to ACE2 leads to ACE2 internalization into endosomes (5) and because ACE2 can be degraded in lysosomes (22), next we determined whether lysosomes are involved in DiMC-induced ACE2 down regulation. DiMC-induced decreases of ACE2 protein expression levels were lysosome dependent because inhibiting vacuolar-ATPase with bafilomycin A1 (100 nM) prevented DiMC (4 μM for 24 h)-induced decreases of ACE2 protein levels in Calu-3 cells (Figure 2C). These findings suggest that DiMC enhanced lysosome degradation of ACE2.

### DiMC Enhanced Lysosome Function

**FIGURE 3 |:**
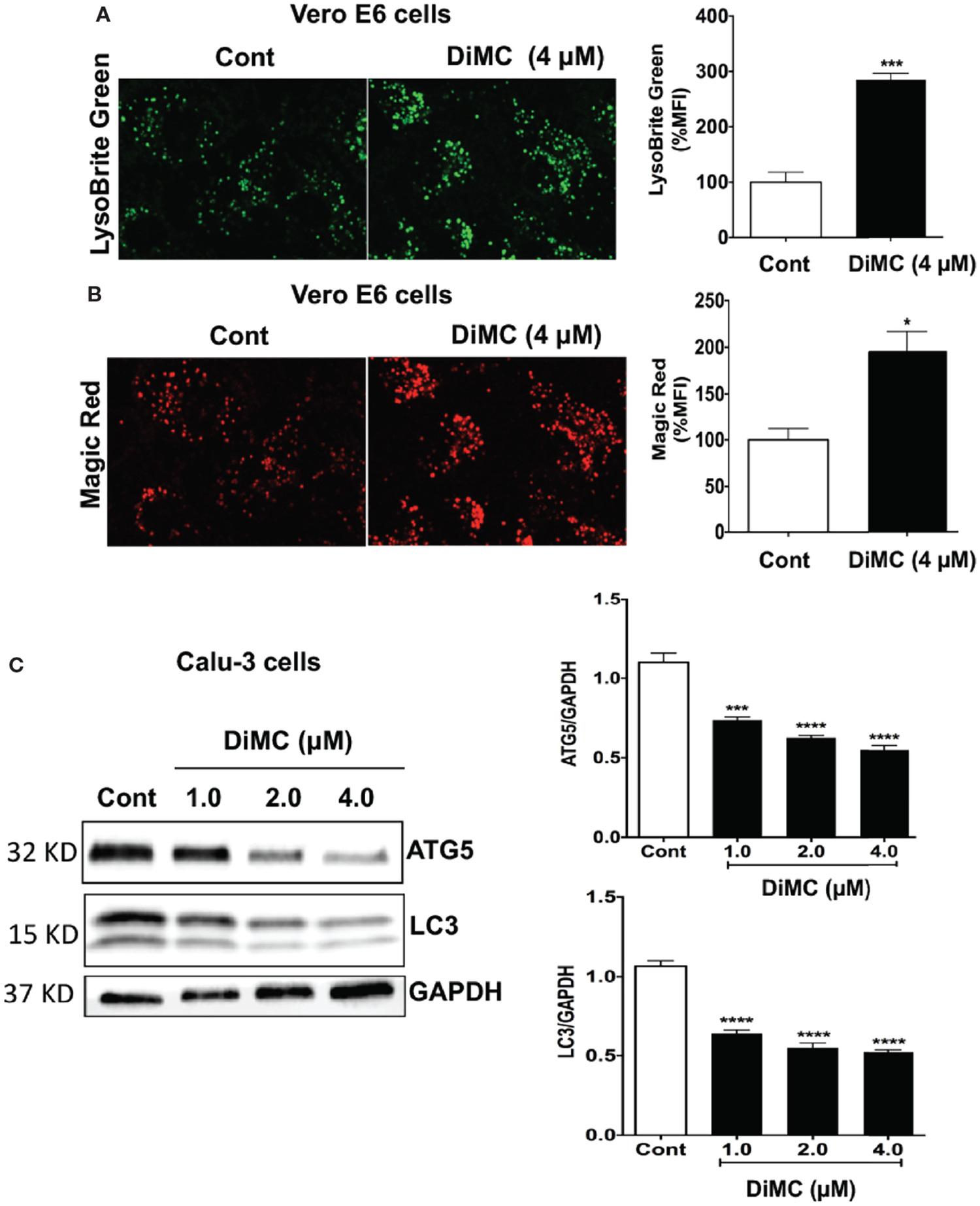
DiMC increased endolysosome acidification, increased cathepsin B activity and decreased autophagy: **(A)** Endolysosome pH was estimated by a pH sensitive LysoBrite green dye in Vero E6 cells. DiMC (4 μM for 18 h) increased fluorescence intensity of LysoBrite (n = 3, *p<0.05); increases in fluorescence intensity indicates decreases (acidification) in endolysosome pH. **(B)** DiMC (4 μM for 18 h) significantly enhanced the activity of cathepsin B as indicated by increased mean fluorescence intensity of Magic Red in Vero E6 cells. (n = 3, ***p <0.001). **(C)** DiMC (1.0, 2.0, and 4.0 μM for 18 h) significantly decreased protein expression levels of the autophagy markers ATG5 and LC3B in Calu-3 cells (n = 3, ***p <0.001, ****p <0.0001).

Curcumin enters lysosomes (23) and increases lysosome acidification and enzyme activity (24). Similarly, monocarbonyl analogs of curcumin promote lysosome biogenesis (25). Accordingly, we next determined effects of DiMC on lysosome functions including levels of pH, cathepsin B activity, and levels of autophagy markers. DiMC (4 μM for 18 h) significantly (p<0.001) increased fluorescence intensity of LysoBrite in Vero E6 cells; an indication of increased acidification (Figure 3A). Because lysosome enzyme activity is related to pH, we next determined effects of DiMC on activity of lysosomal enzymes with Magic-red (cathepsin B). DiMC (4 μM for 18 h) significantly (p<0.05) enhanced the activity of cathepsin B as indicated by increased mean fluorescence intensity of Magic Red in Vero E6 cells (Figure 3B). Lysosomes are also important for degradation of both extracellular cargos delivered *via* endocytosis and intracellular cargos delivered *via* autophagy. Accordingly, we next determined the extent to which DiMC affected lysosome degradation of intracellular cargo *via* autophagy. DiMC (1 to 4 μM for 18 h) significantly decreased protein levels of autophagy associated LC3B and autophagy-initiating ATG5 in Calu-3 cells (Figure 3C). Together these findings suggest that DiMC enhances lysosome degradative capabilities.

### DiMC Decreased Cellular Levels of Pseudo-SARS-CoV-2 and SARS-CoV-2 S1 Proteins

**FIGURE 4 |:**
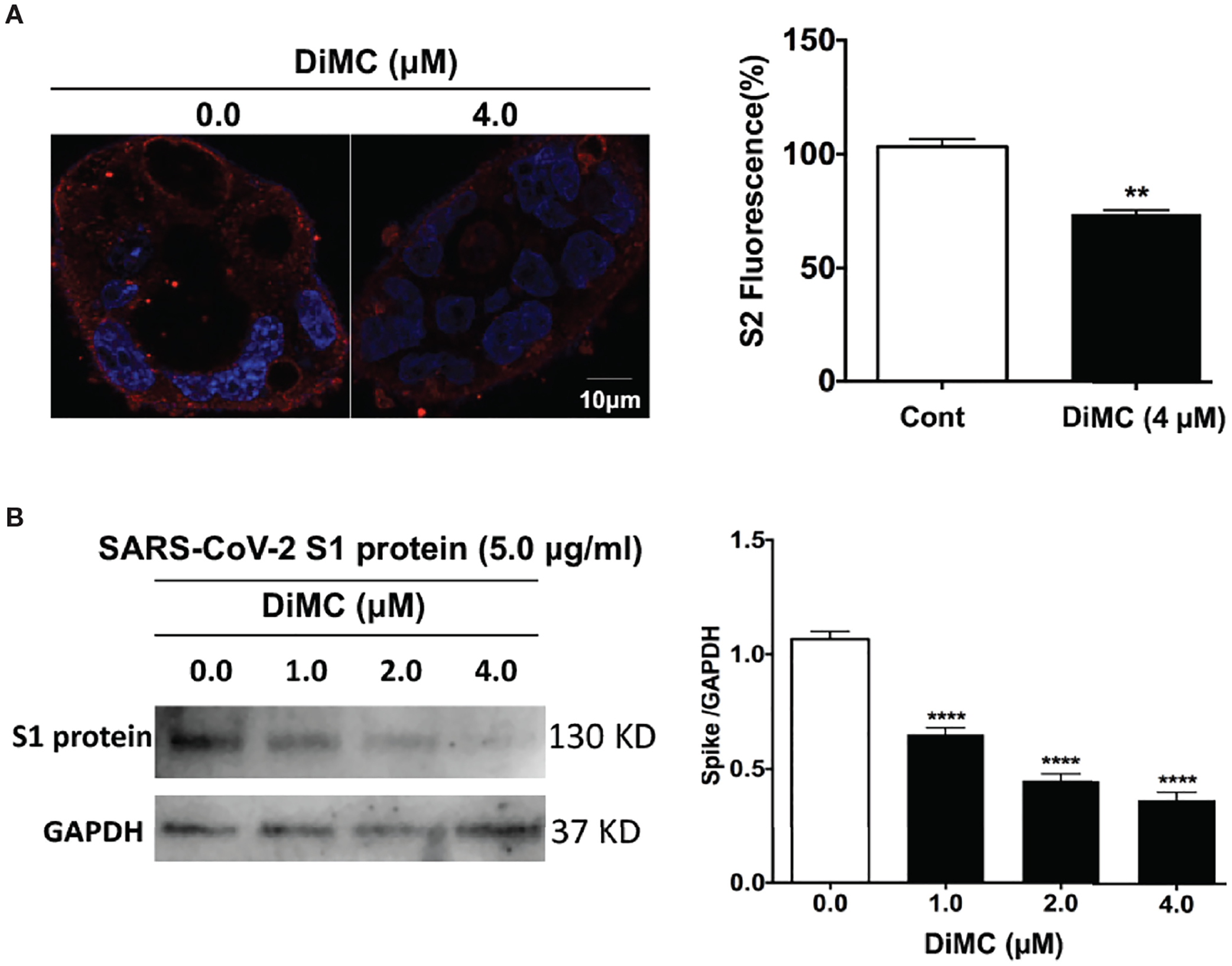
DiMC decreased cellular levels of pseudo-SARS-CoV-2 and SARS-CoV-2 S1 proteins: **(A)** In pseudo-SARS-CoV-2 infected Calu-3 cells, DiMC (4.0 μM for 18 h) decreased spike protein expression levels in Calu-3 cells (bar = 10 μm; n = 3, **p <0.01). **(B)** In Calu-3 cells treated with SARS-CoV-2 spike protein (5.0 μg/ml), DiMC (1.0, 2.0, 4.0 μM for 18 h) significantly decreased cellular levels of SARS-CoV-2 spike protein (n = 3, ****p <0.0001).

We next explored the possibility that DiMC promoted the degradation of SARS-CoV2. DiMC (4 μM for 18 h) significantly (p<0.01) decreased cellular levels of S2 protein in Calu-3 cells treated with pseudo-SARS-CoV-2 (Figure 4A). These findings suggest that DiMC prevented the formation of S2-mediated viral envelope fusion with the endolysosome membrane possibly by enhanced degradation of SARS-CoV-2. To further test this possibility, we treated cells with recombinant SARS-CoV-2 S1 proteins (5 μg/ml), which possess ACE2 receptor binding domains and can be internalized (26). DiMC (4 μM for 18 h) significantly (p<0.0001) decreased cellular levels S1 proteins (Figure 4B). In cultured media, we did not find detectable levels of S1 protein by immunoblotting, indicating all externally added S1 protein was internalized (data not shown).

### Lysosome Acidifying Agents Blocked S-Mediated Entry of Pseudo-SARS-CoV-2

**FIGURE 5 |:**
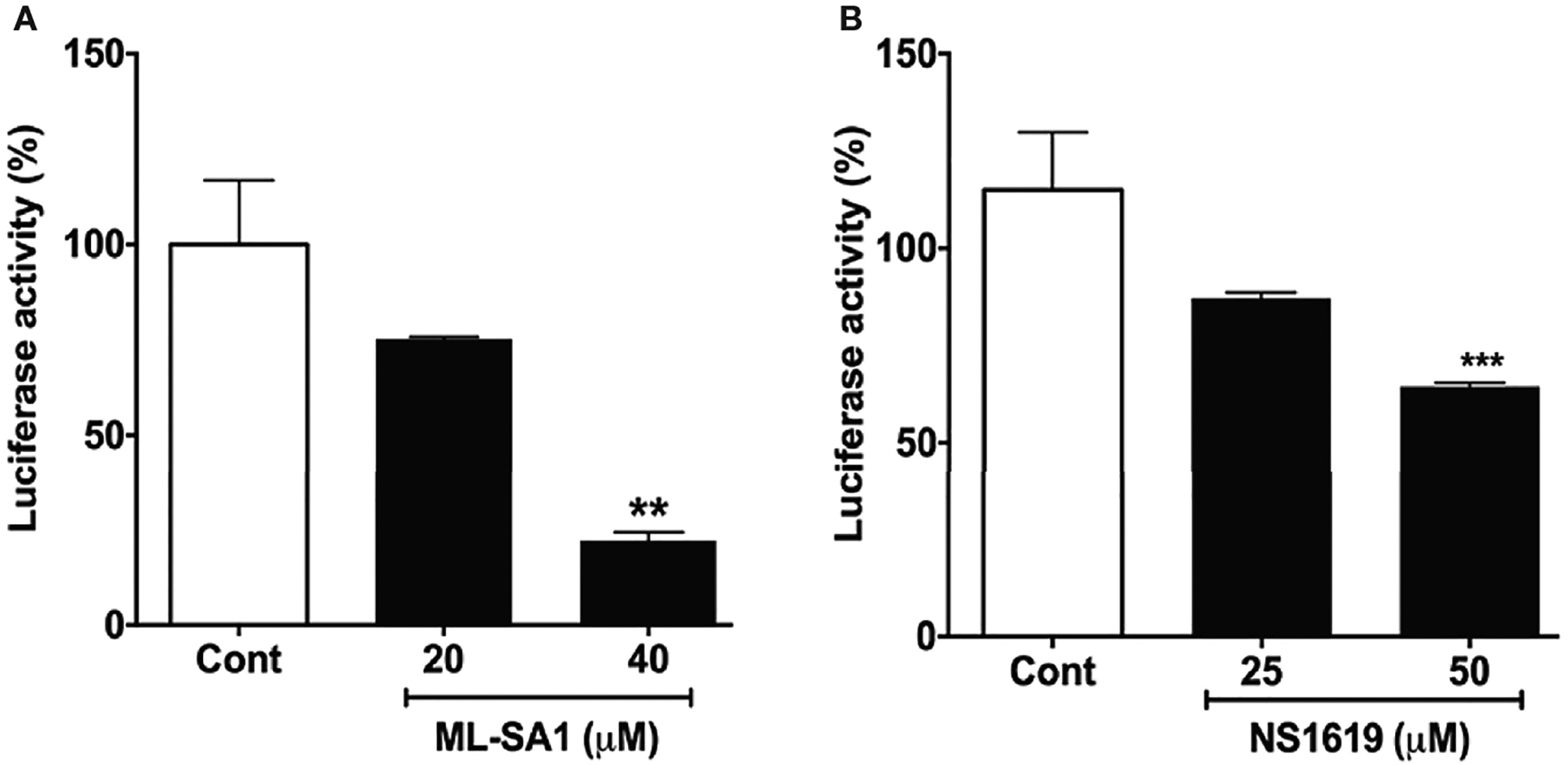
ML-SA1 and NS1619 attenuated SARS-CoV-2 spike protein-mediated entry of pseudo-SARS-CoV-2: **(A)** ML-SA1 treatment (40 μM for 18 h) significantly attenuated SARS-CoV-2 spike protein-mediated entry of pseudo-SARS-CoV-2 as indicated by decreases in luciferase activity in Calu-3 cells (n = 3, **p<0.01). **(B)** NS1619 treatment (50 μM for 18 h) significantly attenuated SARS-CoV-2 spike protein-mediated entry of pseudo-SARS-CoV-2 as indicated by decreases in luciferase activity in Calu-3 cells (n = 3, ***p<0.001).

Next, we determined the extent to which additional agents capable of acidifying lysosomes could affect entry of pseudo-SARS-CoV-2. We reported previously that the TRPML1 agonist ML-SA1 and the BK channel activator NS1619 both acidify lysosomes (27). ML-SA1 (20 and 40 μM for 18 h) and NS1619 (25 and 50 μM for 18 h) significantly attenuated the entry of pseudo-SARS-CoV-2 in Calu-3 cells as indicated by decreases in luciferase activity (Figure 5).

## DISCUSSION

The main findings reported here are that (1) DiMC blocked the entry of pseudo-SARS-CoV-2 into cells, (2) DiMC acidified endolysosomes, enhanced lysosome function and promoted the degradation of ACE2 receptors as well as internalized pseudo-SARS-CoV-2 and SARS-CoV-2 S1 proteins, and (3) that additional lysosome acidifying agents also blocked the spike protein-mediated entry of pseudo-SARS-CoV-2.

A hallmark feature of lysosomes is their acidic luminal pH (28–31), which is critical for the optimal activity of up to 60 different pH-sensitive hydrolytic enzymes including proteases, lipases and nucleases (32). As such, lysosomes are important for degradation of extracellular macromolecules *via* endocytosis and intracellular macromolecules and organelles *via* autophagy.

Endolysosomes (endosomes, lysosomes, and autolysosomes) form an integral part of the SARS-CoV-2 infection process including viral entry into (5, 33) and egress from host cells (34). S protein, a transmembrane glycoprotein that forms homotrimers, is composed of S1 and S2 subdomains (35); S1 is responsible for binding to receptors (ACE2, neurophilin-1, and others) on host cells, whereas S2, the transmembrane portion of S protein, is responsible for fusion of viral membrane with host cell membranes. The strong binding of S protein with ACE2 (36), which is abundantly expressed in lung alveolar epithelial cells (37), allows the formation of stable association of SARS-CoV-2 with host cell plasma membrane. The cleavage of the S protein between the S1 and S2 domains by various cellular proteases such as TMPRSS2, furin, and cathepsins (3, 4, 20, 38) results in the release of S1 protein and the exposure of S2 protein, which mediates the fusion of the viral membrane and the host cell membrane and subsequent releasing of viral RNA into the cytoplasm of the host (7, 8).

The S2-mediated viral fusion processes could occur at the plasma membrane (39) or within endolysosomes, where viral membrane fuses with the endolysosome membrane following receptor-mediated endocytosis of SARS-CoV-2 (5). In the latter scenario, the initial cleavage of S protein between the S1 and S2 domains would occur in endolysosomes by pH-sensitive proteases such as furin (40) that is internalized (41) and lysosome-resident cathepsins (42). At the beginning of COVID-19 outbreak, pH-dependent SARS-CoV-2 viral entry, as explained in the latter scenario above, provided rationale for testing lysosome de-acidifying agents for the treatment of SARS-CoV-2, such as the use of chloroquine and hydroxychloroquine, both of which are diprotic weak base that accumulate in endolysosomes and neutralize the acidic luminal pH (43, 44). Early *in vitro* evidence indicated that chloroquine and hydroxychloroquine inhibited the ability of SARS-CoV-2 to infect monkey kidney-derived Vero E6 cells (45–47). However, such anti-viral effects of chloroquine and hydroxychloroquine were not replicated in human lung cells (21), which is consistent with clinical studies showing that chloroquine/hydroxychloroquine had no therapeutic effect on COVID-19 patients (48–50) and even with worsened outcomes (51, 52).

Our findings suggest that acidifying endolysosomes represents a promising therapeutic strategy against SAR-CoV-2 infection; acidifying lysosomes would allow complete degradation of internalized SARS-CoV-2 by more than 50 pH-sensitive hydrolytic enzymes presented in lysosomes, thus preventing the formation of S2-mediated viral fusion with endolysosome membranes. Indeed, we demonstrated that DiMC, an analog of a natural polyphenol curcumin (12), blocked S-mediated pseudo-SARS-CoV-2 viral entry in both Calu-3 cells and Vero E6 cells. Furthermore, other lysosome acidifying agents including the TRPML1 agonist ML-SA1 and the BK channel activator NS1619 (27) also attenuated S-mediated entry of pseudo-SARS-CoV-2. Validation of the anti-SARS-CoV-2 potential of DiMC and other lysosome acidifying agents using live SARS-CoV-2 virus and using other cell models especially in primary cells is warranted. In addition, DiMC exhibits anti-inflammatory activities including reducing IL-6 levels (17, 53); such immune modulatory properties of DiMC could counteract inflammatory responses induced by SARS-CoV-2 (54, 55) and warrant further exploration.

Mechanistically, DiMC acidified endolysosomes, enhanced degradation capabilities of lysosomes, and promoted the degradation of ACE2, internalized pseudo-SARS-CoV-2, and internalized SARS-CoV-2 S1 proteins. Currently, it is not known how DiMC acidifies endolysosomes. Based on findings that a fraction of curcumin enters endolysosomes (23), it is possible that DiMC mediates endolysosome enhancing effects *via* unknown actions within endolysosomes. Alternatively, DiMC, similar to that of curcumin or curcumin analog (24, 25, 56), could enhance lysosome biogenesis *via* activating transcription factor EB (TFEB), a master regulator of autophagy and lysosomal biogenesis.

Not without caveat and limitation, our findings suggest that acidifying endolysosomes and enhancing lysosome degradation capabilities of host cells by DiMC and other lysosome acidifying agents have promising anti-SARS-CoV-2 potential. If validated with live SARS-CoV-2 virus, such findings will provide rationale for developing DiMC and other lysosome acidifying agents as effective therapeutic strategies against SARS-CoV-2 and COVID-19.

## Data Availability

The original contributions presented in the study are included in the article/supplementary material. Further inquiries can be directed to the corresponding author.
